# Assessment of Serum Zinc Levels of Patients with Thalassemia Compared to Their Siblings

**DOI:** 10.1155/2014/125452

**Published:** 2014-08-14

**Authors:** Mohamed El Missiry, Mohamed Hamed Hussein, Sadaf Khalid, Naila Yaqub, Sarah Khan, Fatima Itrat, Cornelio Uderzo, Lawrence Faulkner

**Affiliations:** ^1^Cure2Children Foundation, Via Marconi 30, 50131 Florence, Italy; ^2^Children's Hospital Pakistan Institute of Medical Sciences, Islamabad, Pakistan

## Abstract

Zinc (Zn) is essential for appropriate growth and proper immune function, both of which may be impaired in thalassemia children. Factors that can affect serum Zn levels in these patients may be related to their disease or treatment or nutritional causes. We assessed the serum Zn levels of children with thalassemia paired with a sibling. Zn levels were obtained from 30 children in Islamabad, Pakistan. Serum Zn levels and anthropometric data measures were compared among siblings. Thalassemia patients' median age was 4.5 years (range 1–10.6 years) and siblings was 7.8 years (range 1.1–17 years). The median serum Zn levels for both groups were within normal range: 100 *μ*g/dL (10 *μ*g/dL–297 *μ*g/dL) for patients and 92 *μ*g/dL (13 *μ*g/dL–212 *μ*g/dL) for siblings. There was no significant difference between the two groups. Patients' serum Zn values correlated positively with their corresponding siblings (*r* = 0.635, *P* < 0.001). There were no correlations between patients' Zn levels, height for age Z-scores, serum ferritin levels, chelation, or blood counts (including both total leukocyte and absolute lymphocyte counts). Patients' serum Zn values correlated with their siblings' values. In this study, patients with thalassemia do not seem to have disease-related Zn deficiency.

## 1. Introduction

Zinc (Zn) is an essential element for cell growth, differentiation, and survival. It is a structural element of many proteins [1]. Zinc affects growth in children. It is known that adequate zinc levels in the body are essential for maintaining suitable levels of growth hormone and insulin-like growth factor in the body [2]. Impairment of zinc levels will consequently lead to growth hormone decrease. Zinc supplement is given to children on growth hormone replacement therapy. In addition, zinc is important for nucleic acid synthesis, cell division, and metabolism of lipids, proteins and carbohydrates. It is also essential in bone homeostasis and bone growth as well as in the maintenance of connective tissues. Decreased Zn may compromise growth and immune functions [2, 3].

Zn is known to be important for the integrity of the immune system, although its role and mechanism of action are not fully understood [1, 4–6]. Zn deficiency affects the adaptive immune system and results in thymus atrophy, lymphopenia, and impaired lymphocyte function [4, 7, 8].

Zinc deficiency is prevalent in children of developing countries where food is often vegetable-based and rarely includes animal products. Zinc is easily absorbed with animal proteins, while excess plant meals lead to decreased zinc absorption due to its binding to phytates [9, 10]. In such countries, Zn deficiency results in growth retardation, hypogonadism, and increased mortality and morbidly from infection-related diarrhea and pneumonia due to compromised immune function [4, 9].

Despite deficits of several specific micronutrients reported in children with thalassemia major, Zn studies yielded conflicting results [7, 11, 12]. Several factors contribute to zinc deficiency in thalassemia. One of these most important factors is chelation therapy. Chelators, namely, deferoxamine and deferiprone, may contribute to Zn deficiency in thalassemia as they tend to eliminate positive divalent ions, like iron and Zn, into urine [7, 13, 14]. On the other side, some studies showed no significant correlation between zinc level and short stature, serum ferritin level, desferrioxamine dose, age at first blood transfusion, and chelation therapy [7]. Zinc can be normal in some patients especially those who are on regular blood transfusions [7, 11, 12]. What is notable that these studies were performed on adequately treated patients subjects which is not the case in many thalassemia affected areas where access to treatment is not always possible.

In the present study, we aimed to assess serum Zn levels in patients with thalassemia and their siblings in a lower middle income country, namely, Pakistan (http://data.worldbank.org/country/pakistan), to determine whether Zn deficiency is present and, if so, if it is related to the disease per se, the use of chelation or to nutritional factors.

## 2. Patients and Methods

The present study was performed at the Children's Hospital of the Pakistan Institute of Medical Sciences (PIMS), Islamabad, Pakistan, between June 2009 and February 2012. A total of 30 patients with *β*-thalassemia major and 30 siblings were included. Parental informed consent was obtained. The following data were obtained from the patients' clinical file records: blood transfusion history, last ferritin measurement, onset of chelation therapy and type of chelation used, and infection profile. In addition to serum Zn levels, anthropometric measures such as height, weight, and body mass index (BMI) (BMI = weight (kg)/height^2^ (m^2^) were obtained).

Sampling and processing: Three mL of peripheral venous blood was withdrawn from each patient and sibling in the early morning, three hours before having breakfast. The samples were left for 20 minutes to clot at room temperature and then centrifuged at 2000 ×g for 10 minutes, and sera were separated and put into aliquots which were stored at −70°C till they were analyzed by atomic absorption spectrometer (AAS; AA300). Zn normal values were estimated to lie between 65 and 120 *μ*g/dL [15].

Statistical analyses were performed by parametric single and paired *t*-test (after the run of normality test to check that data is normally distributed) and Pearson's correlation coefficient test. A *P* value ≤ 0.05 was considered to be statistically significant.

## 3. Results

**Figure 1:**
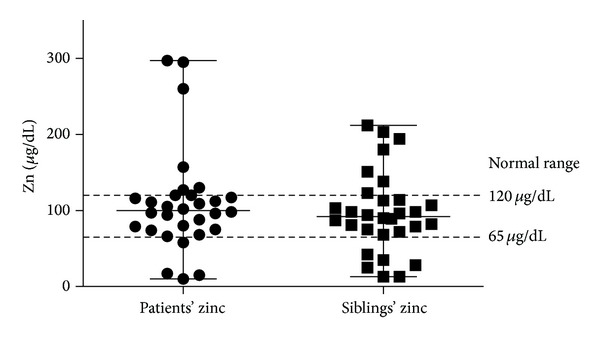
Comparison between serum Zn levels for patients and their corresponding siblings. Median levels were within normal range: 100 *μ*g/dL (10 *μ*g/dL–297 *μ*g/dL) for patients and 92 *μ*g/dL (13 *μ*g/dL–212 *μ*g/dL) for siblings.

**Figure 2:**
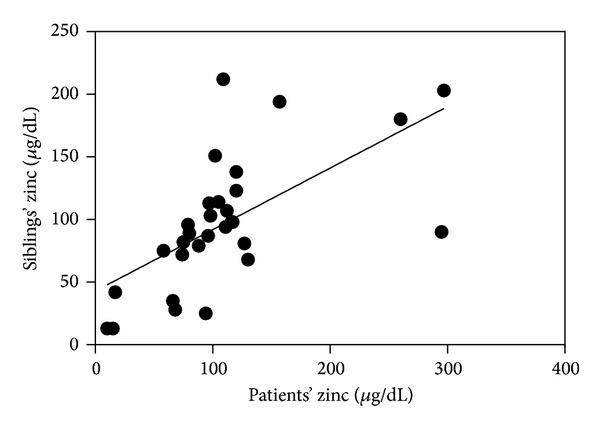
Correlation between patients' serum Zn values. Levels correlated positively with corresponding (*r* = 0.635, *P* < 0.001).

Median patient age was 4.5 years (ranging from 1 to 10.6 years) and median sibling age was 7.3 years (ranging from 1.1 to 17 years). Patients' serum Zn ranged from 10 *μ*g/dL to 297 *μ*g/dL (median 100 *μ*g/dL), while siblings' serum Zn ranged from 13 *μ*g/dL to 212 *μ*g/dL (median 92 *μ*g/dL) (Figure 1). There were no significant differences in Zn levels between the patients and their corresponding siblings (*P* = 0.19) on matched pair analysis (Table 1). However, it was found that Zn levels were significantly correlated between patients with their corresponding siblings (*r* correlation coefficient = 0.63, *P* < 0.0001) (Figure 2).

After measuring the height for age *Z*-scores for the 30 patients with thalassemia, it was found that heights ranged between −4.2 at minimum and 1.09 at maximum (median = −1.5). Height for age *Z*-scores and Zn levels did not correlate (*r* = 0.05).

For siblings, the median height for age *Z*-scores was −1.2 and ranged from −3.78 to 2.6 (median = −1.2). A comparison of patient height for age *Z*-scores with corresponding values for siblings revealed that patients' *Z*-scores levels were significantly lower than corresponding siblings (*P* = 0.02). Correlation between the two groups was weakly positive (*r* = 0.4), and pairing between the two groups was statistically significant (*P* = 0.01).

The median patient serum ferritin level was 2065 ng/mL (range: 5475 ng/mL–81.15 ng/mL) and showed no correlation with patients' Zn level (*P* = 0.7).

Regular chelation therapy was used by 14 patients: 7 cases were on deferasirox and 7 cases on desferrioxamine, while 16 cases received no chelation therapy. No significant difference in Zn levels was found between chelated and nonchelated groups (median zinc levels were 103.5 *μ*g/dL and 96 *μ*g/dL for chelated and nonchelated patients, respectively, *P* = 0.63). There was also no significant difference in zinc values between patients on desferrioxamine and those on deferasirox (median zinc levels were 96 *μ*g/dL and 102 *μ*g/dL for patients with desferrioxamine and deferasirox, respectively, *P* = 0.37).

Absolute lymphocytic count (ALC) ranged between 817 and 9040 microL, with a median of 3882 microL. No correlation was found between patients' Zn values and ALC (*r* = 0.36).

## 4. Discussion

Zinc is an essential element for growth and immunity. In this study we aimed to compare serum Zn levels between thalassemia patients and their healthy siblings as to assess whether a possible deficiency is influenced by the disease itself or by nutritional and familial/environmental causes.

Patients' and siblings' Zn median values were within the normal range (median values were 100 *μ*g/dL and 92 *μ*g/dL for patients and siblings, resp.) with no significant difference (patients' serum Zn median value was 100 *μ*g/dL versus 92 *μ*g/dL for siblings) (Figure 1). This finding is in agreement with studies by Rea et al. (1984) [12] and Donma et al. (1990) [11] who noted that the serum Zn levels of patients with thalassemia can be higher than normal [11, 12]. Among the 30 siblings in this study, 18 were carriers for beta thalassemia and 12 were not with no significant differences between the two groups, suggesting that Zn status is not related to thalassemia—the disease itself or trait. The patients' Zn values in this study correlated significantly with corresponding siblings (*r* correlation coefficient = 0.63, *P* < 0.0001) (Figure 2) suggesting that Zn level was not influenced by thalassemia or its treatment [7], but rather seems more likely related to familial factors either genetic or nutritional/environmental [7].

No correlation between Zn values and growth (height for age *Z*-scores) was observed in our patients. Similar results have been found in several other studies; thus, it is assumed that a Zn deficiency is not related to short stature [7, 16]. However, in a study by Kyriakou and Skordis (2009) [17], the authors proposed that Zn deficiency could be a concomitant factor for growth failure among patients with thalassemia [17]. As patients were found to have significant lower height-for-age compared to their siblings, however there are other concomitant variables such as chronic anemia, iron overload-related endocrine problems, and impaired bone growth which play an important role [18–20]. In our study zinc levels did not seem to differ among siblings suggesting that Zn deficiency may not play a significant role in growth differences often observed between thalassemic children and their brothers or sisters.

It appears that elevated ferritin levels are inversely related to Zn levels so that as ferritin increases, Zn decreases. However, in this study, the correlation was not statically significant. Decreased zinc levels or increased ferritin values have been previously reported [7, 21] and might be explained by inadequacy of clinical care and proper management affecting independently both ferritin and nutritional Zn levels.

With the limitation of a small sample size, in our study chelation therapy did not seem to affect zinc levels. Deferoxamine and deferiprone have been reported to also chelate and eliminate zinc into urine, while for deferasirox, which has a lower affinity for divalent zinc, this seems not to be the case [7, 13, 14].

Several studies have assumed that Zn is important to maintain intact lymphocytic function and counts [4, 7]. Fraker and King (2004) [8] found that a Zn deficiency led to lymphopenia [8]. In conclusion, this study showed that patients with thalassemia do not seem to be prone to Zn deficiency. Patients' serum Zn values correlated with their sibling suggesting that serum Zn levels are possibly more influenced by familial and environmental factors rather than by thalassemia per se or its treatment.

## Figures and Tables

**(a):** 

Patient number	Zinc level (*μ*g/dL)	Sex	Age (years)	Height (cm)	Height-age *z*-score	Ferritin (ng/mL)	Chelation	ALC (microL)
1	94	m	2.6	84	−1.33	694	None	6325
2	58	m	2.9	92	−0.97	2527	None	6200
3	68	f	5.3	100	−2.29	3004	Deferasirox	3903
4	297	m	2.5	92	−0.04	785	None	3891
5	130	m	1	72	−2	641	None	7956
6	66	m	5.5	106	−1.51	2129	Deferoxamine	2156
7	102	f	2.5	91	−1	2000	Deferasirox	8024
8	80	f	8.8	122	−1.57	3513	Deferoxamine	2340
9	109	m	3.8	88	−4	1551	None	7526
10	157	m	4.4	106	−1	2425	Deferoxamine	3872
11	120	m	8.2	115	−2.31	1502	Deferoxamine	3008
12	74	m	2.5	89	−1.17	616	None	4884
13	127	m	5.4	105	−1.48	3810	None	3570
14	75	f	4	95	−1.79	1150	None	1700
15	117	f	4.6	111	0.92	1804	Deferasirox	1218
16	295	m	1.9	81	−1.6	850	None	9040
17	88	m	10.6	131	−1.5	5475	Deferasirox	1160
18	116	f	4	95	−2	2836	None	2813
19	17	m	4.7	113	1.09	658	None	2592
20	111	m	5.6	109	−1.18	2411	Deferoxamine	5002
21	112	m	7.1	114	−1.53	1951	None	1353
22	10	m	1.6	72	−4.2	81.15	None	817
23	79	m	2.2	83	−1.9	2621	None	5490
24	15	f	5	110	0.14	5036	Deferoxamine	1980
25	97	f	10	124	−2.32	706	Deferasirox	4312
26	96	f	10.4	139	−0.32	4487	Deferoxamine	3062
27	120	m	4.5	106	−0.33	5054	None	3180
28	105	f	7.4	117	−1.41	1760	Deferasirox	4260
29	98	m	1.3	75	−1.9	3812	None	5535
30	260	m	4.4	98	−2.01	4100	Deferasirox	3944

**(b):** 

Sibling number	Zinc level (*μ*g/dL)	Sex	Age (years)	Height (cm)	Height-age *z*-score	Sibling carrier status
Sibling of 1	25	f	12.0	143	−1.23	Not a carrier
Sibling of 2	75	m	5.9	128	2.62	Carrier
Sibling of 3	28	f	9.4	127	−1.31	Not a carrier
Sibling of 4	203	m	6.2	108	−1.85	Not a carrier
Sibling of 5	68	f	11.5	136	−1.8	Carrier
Sibling of 6	35	m	2.4	89	−0.48	Carrier
Sibling of 7	151	f	1.1	77	0.74	Not a carrier
Sibling of 8	89	m	4.1	108	0.95	Carrier
Sibling of 9	212	f	8.7	117	−2.24	Carrier
Sibling of 10	194	f	12.4	150	−0.53	Carrier
Sibling of 11	138	m	6.9	110	−2.16	Not a carrier
Sibling of 12	72	m	6.9	119	−0.41	Carrier
Sibling of 13	81	m	1.5	75	−2.5	Not a carrier
Sibling of 14	82	m	12.0	140	−1.26	Carrier
Sibling of 15	98	f	2.6	144	−1.51	Not a carrier
Sibling of 16	90	f	5.3	104	−1.39	Carrier
Sibling of 17	79	f	17.0	149	−2.07	Carrier
Sibling of 18	98	m	14.1	150	−1.8	Not a carrier
Sibling of 19	42	f	9.0	137	0.74	Not a carrier
Sibling of 20	94	f	7.5	118	−1.03	Not a carrier
Sibling of 21	107	f	11.9	132	−2.7	Carrier
Sibling of 22	13	m	3.2	89	−2.18	Carrier
Sibling of 23	96	m	11.2	134	1.5	Carrier
Sibling of 24	13	f	7.2	124	0.39	Carrier
Sibling of 25	113	m	8.0	106	−3.78	Carrier
Sibling of 26	87	f	2.4	90	−0.01	Carrier
Sibling of 27	123	f	7.2	130	1.45	Carrier
Sibling of 28	114	f	1.2	73	−1.1	Carrier
Sibling of 29	103	f	9.2	140	0.98	Not a carrier
Sibling of 30	180	f	12.0	138	−1.94	Not a carrier

## Abbreviations

ALC: Absolute lymphocytic count
BMI: Body mass index
CMV: Cytomegalovirus
IL-2: Interleukin 2
IL-10: Interleukin 10
Treg: T regulatory cells
Zn: Zinc.

## References

[1] Hirano T, Murakami M, Fukada T, Nishida K, Yamasaki S, Suzuki T (2008). Roles of zinc and zinc signaling in immunity: zinc as an intracellular signaling molecule. *Advances in Immunology*.

[2] MacDonald RS (2000). The role of zinc in growth and cell proliferation. *Journal of Nutrition*.

[3] Brandão-Neto J, Stefan V, Mendonça BB, Bloise W, Castro AVB (1995). The essential role of zinc in growth. *Nutrition Research*.

[4] Yu M, Lee W-W, Tomar D (2011). Regulation of T cell receptor signaling by activation-induced zinc influx. *The Journal of Experimental Medicine*.

[5] Kadrmas JL, Beckerle MC (2004). The LIM domain: from the cytoskeleton to the nucleus. *Nature Reviews Molecular Cell Biology*.

[6] Moshtaghi-Kashanian G, Gholamhoseinian A, Hoseinimoghadam A, Rajabalian S (2006). Splenectomy changes the pattern of cytokine production in *β*-thalassemic patients. *Cytokine*.

[7] Mehdizadeh M, Zamani G, Tabatabaee S (2008). Zinc status in patients with major *β*-thalassemia. *Pediatric Hematology and Oncology*.

[8] Fraker PJ, King LE (2004). Reprogramming of the immune system during zinc deficiency. *Annual Review of Nutrition*.

[9] Yakoob MY, Theodoratou E, Jabeen A (2011). Preventive zinc supplementation in developing countries: impact on mortality and morbidity due to diarrhea, pneumonia and malaria. *BMC Public Health*.

[10] Gibson RS, Ferguson EL (1998). Nutrition intervention strategies to combat zinc deficiency in developing countries. *Nutrition Research Reviews*.

[11] Donma O, Gunbey S, tas Donma MAMM (1990). Zinc, copper, and magnesium concentrations in hair of children from southeastern Turkey. *Biological Trace Element Research*.

[12] Rea F, Perrone L, Mastrobuono A, Toscano G, D'Amico M (1984). Zinc levels of serum, hair and urine in homozygous beta-thalassemic subjects under hypertransfusional treatment. *Acta Haematologica*.

[13] Galanello R (2007). Deferiprone in the treatment of transfusion-dependent thalassemia: a review and perspective. *Therapeutics and Clinical Risk Management*.

[14] Cappellini MD (2007). Exjade (deferasirox, ICL670) in the treatment of chronic iron overload associated with blood transfusion. *Therapeutics and Clinical Risk Management*.

[15] Hambidge M (2000). Human zinc deficiency. *Journal of Nutrition*.

[16] Fuchs GJ, Tienboon P, Linpisarn S (1996). Nutritional factors and thalassaemia major. *Archives of Disease in Childhood*.

[17] Kyriakou A, Skordis N (2009). Thalassaemia and aberrations of growth and puberty. *Mediterranean Journal of Hematology and Infectious Diseases*.

[18] de Sanctis V, Eleftheriou A, Malaventura C (2004). Prevalence of endocrine complications and short stature in patients with thalassaemia major: a multicenter study by the Thalassaemia International Federation (TIF). *Pediatric Endocrinology Reviews*.

[19] Theodoridis C, Ladis V, Papatheodorou A (1998). Growth and management of short stature in thalassaemia major. *Journal of Pediatric Endocrinology and Metabolism*.

[21] Mahyar A, Ayazi P, Pahlevan AA, Mojabi H, Sehhat MR,  Javadi A (2010). Zinc & copper status in children with Beta-thalassemia major. *Iranian Journal of Pediatrics*.

